# Supplementary material for: Subcellular Targeting of Plant Sucrose Transporters Is Affected by Their Oligomeric State
Source: Plants (Basel). 2020 Jan 27;9(2):158. doi: 10.3390/plants9020158 (PMC7076641; doi:10.3390/plants9020158)
Supplement: Supplementary file 1 [file plants-09-00158-s001.pptx]

## Slide 1
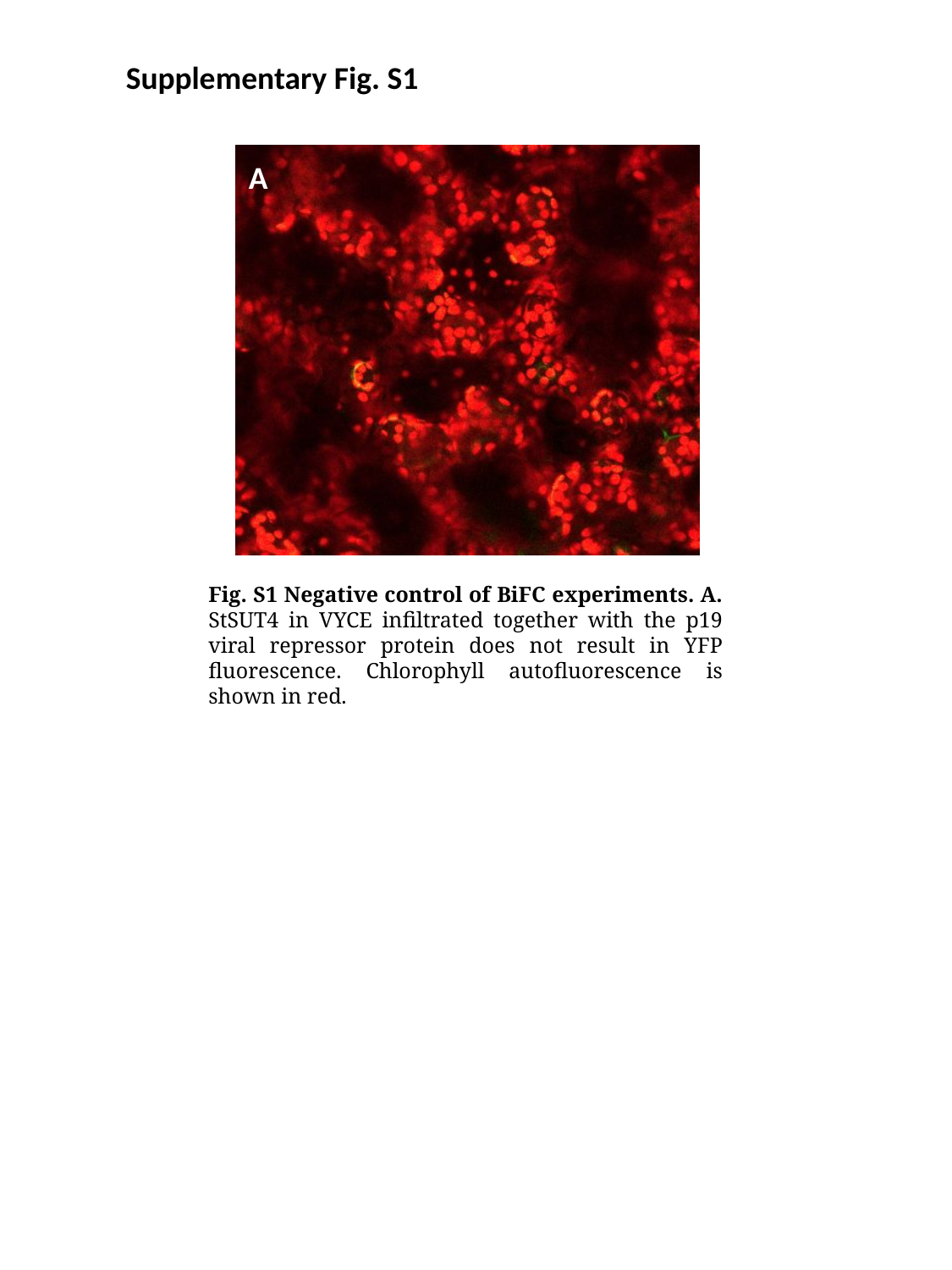

Supplementary Fig. S1
A
Fig. S1 Negative control of BiFC experiments. A. StSUT4 in VYCE infiltrated together with the p19 viral repressor protein does not result in YFP fluorescence. Chlorophyll autofluorescence is shown in red.

## Slide 2
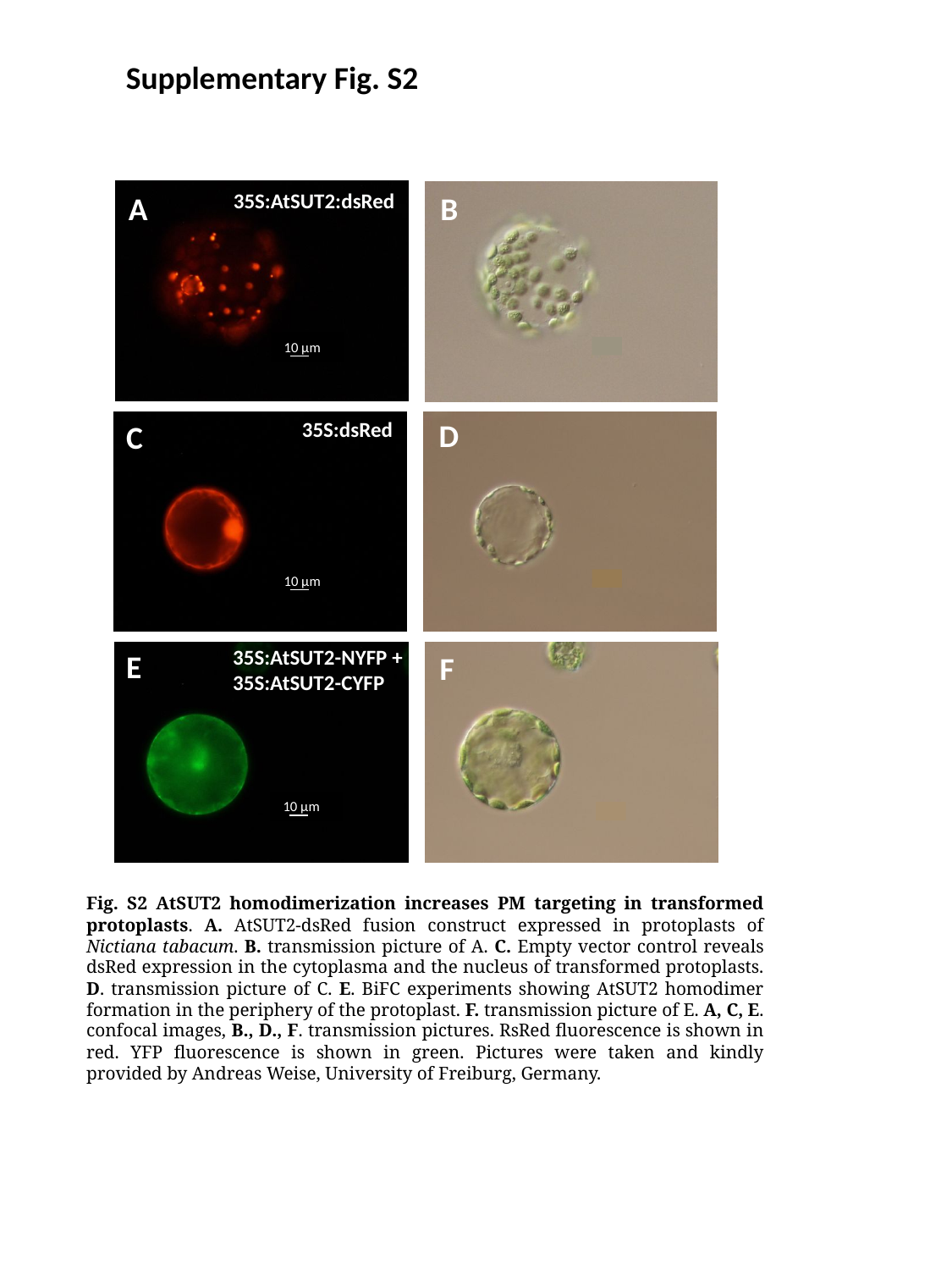

Supplementary Fig. S2
35S:AtSUT2:dsRed
A
B
10 µm
35S:dsRed
D
C
10 µm
35S:AtSUT2-NYFP +
35S:AtSUT2-CYFP
E
F
10 µm
Fig. S2 AtSUT2 homodimerization increases PM targeting in transformed protoplasts. A. AtSUT2-dsRed fusion construct expressed in protoplasts of Nictiana tabacum. B. transmission picture of A. C. Empty vector control reveals dsRed expression in the cytoplasma and the nucleus of transformed protoplasts. D. transmission picture of C. E. BiFC experiments showing AtSUT2 homodimer formation in the periphery of the protoplast. F. transmission picture of E. A, C, E. confocal images, B., D., F. transmission pictures. RsRed fluorescence is shown in red. YFP fluorescence is shown in green. Pictures were taken and kindly provided by Andreas Weise, University of Freiburg, Germany.
